# Cerebrospinal Fluid Analysis in Patients with Post-neurosurgical Procedures: Meningitis vs. Non-meningitis

**DOI:** 10.30699/ijp.2024.2019741.3240

**Published:** 2024-07-24

**Authors:** Mehdi Zeinalizadeh, Maryam Shadkam, Pegah Afarinesh Khaki, Alireza Abdollahi, Masoumeh Douraghi, Mohammadreza Salehi

**Affiliations:** 1 Department of Neurological Surgery, Imam Khomeini Hospital, Tehran University of Medical Sciences, Tehran, Iran; 2 Department of Infectious Diseases, Imam Khomeini Hospital Complex, Tehran University of Medical Sciences, Tehran, Iran; 3 Central Laboratory of Imam Khomeini Hospital Complex, Tehran University of Medical Sciences, Tehran, Iran; 4 Department of Pathology, Imam Khomeini Hospital Complex, Tehran University of Medical Sciences, Tehran, Iran; 5 Medical Bacteriology Section, Department of Pathobiology, School of Public Health. Tehran University of Medical Sciences, Tehran, Iran; 6 Research Center for Antibiotic Stewardship & Antimicrobial Resistance, Department of Infectious Diseases, Imam Khomeini Hospital Complex, Tehran University of Medical Sciences, Tehran, Iran

**Keywords:** Cerebrospinal fluid (CSF) analysis, CSF glucose, CSF leukocyte count, Post-neurosurgical meningitis

## Abstract

**Background & Objective:**

Cerebrospinal fluid (CSF) analysis is helpful in the diagnosis of infections of the central nervous system (CNS), especially after neurosurgical procedures. This study aimed to evaluate the diagnostic value of CSF markers for diagnosis of post-neurosurgical meningitis (PNM).

**Methods:**

Patients with neurosurgical procedures whose CSF was obtained for any reason (meningitis and non-meningitis) during 2020 and 2022, at Imam Khomeini Hospital Complex, Tehran, Iran, were included. Serum and CSF lactate dehydrogenase (LDH), glucose, protein, white blood cells (WBC), red blood cells (RBC), and CSF/serum glucose and LDH ratio were compared between the patients who were diagnosed with PNM and those without meningitis.

**Results:**

A total of 115 patients were included, of whom 23 patients were diagnosed with PNM and 92 with non-meningitis. No significant differences were observed in patients’ age, gender, and underlying diseases between the two groups. Findings showed a significantly (*P*=0.029) lower level of the mean CSF glucose (59.5 mg/dL ±33.9) in patients with meningitis than in patients without meningitis (76.8 mg/dL ± 37.5). The mean CSF/serum glucose ratio was 43.7% in the meningitis group and 56.3% in the non-meningitis group (*P*=0.008). The mean WBC count and neutrophil dominance were significantly higher in the meningitis group. No significant differences were observed in CSF LDH, Protein, and RBC between the two groups.

**Conclusion:**

A CSF glucose level of less than 60 mg/dL, a CSF/serum glucose ratio of less than 0.44, and a higher CSF WBC and neutrophil count can help diagnose PNM.

## Introduction

Post-neurosurgical meningitis (PNM) is a rare but life-threatening surgical complication that can lead to a prolonged hospital stay, high morbidity, and even high mortality rates of 20% to 50% (1, 2). Meningeal inflammation can occur during or after surgery owing to the blood cell lysis, coagulation processes, excisions and sutures, tissue decomposition, or even bacterial inoculation (3, 4). PNM can occur after craniotomy or placement of internal or external ventricular catheters. The reported rates of PNM vary depending on the neurosurgical methods (5). The incidence rate of bacterial meningitis in patients after craniotomy has been reported to be less than 0.5% to more than 8% (6).

PNM is mainly caused by gram-positive organisms colonized on the skin, such as coagulase-negative *Staphylococcus* and *Staphylococcus aureus*. However, PNM caused by aerobic gram-negative bacilli has been increasingly reported (7, 8).

It is essential to distinguish bacterial meningitis from other conditions that can mimic this infectious syndrome. The CSF culture is an important method to diagnose meningitis. However, it is time-consuming and has low sensitivity due to the low number of bacteria in CSF. Moreover, the widespread use of antibiotics before neurosurgical procedures can affect the results of the CSF culture. As a result, 70% of CSF cultures are reported to be negative, making the PNM diagnosis challenging (9-14). There are only a few high-quality studies on the diagnostic methods of PNM. So, current diagnostic approaches are mostly based on low-quality studies and case reports (6). The aim of this study was to compare the CSF parameters in post-neurosurgical patients with meningitis with those without meningitis to evaluate the diagnostic value of CSF markers for the PNM diagnosis.

## Material and Methods

**Study Design**

A retrospective study was conducted on patients with neurosurgical procedures with or without PNM, using their medical records at a referral hospital in Tehran, Iran, between 2020 and 2022.

**Study Population**

The study population was patients with neurosurgical procedures (craniotomy or trans-sphenoidal surgery) whose CSF was obtained for any reason (meningitis and non-meningitis) after the surgery.

Patients receiving antibiotics at the time of the CSF collection, patients with clinical conditions compatible with meningitis but with negative CSF culture and smear, and patients with incomplete medical records were excluded from the study. 

**Data Collection**

The patients’ demographic and clinical data, including age, sex, underlying disease, type of surgery, history of antibiotic and corticosteroid therapy, and laboratory findings were retrieved from the hospital's electronic database. Patients’ CSF and blood samples, which were taken at the same time and within one month after the neurosurgical procedure, were examined for microbiological tests (smear and culture) for the CSF and cell count, differential blood count, and biochemistry (glucose, protein, and lactate dehydrogenase (LDH)) for CSF and blood samples. The laboratory tests were performed according to the standard protocols: Samples were transferred in sterile tubes to the hospital laboratory immediately. Serum and CSF biochemistry analysis and complete blood cell count were performed using Hitachi biochemistry autoanalyzer 7180 (Japan) and Mindray BC-5800 automated hematology analyzer (China), respectively. CSF sediments were cultured on 5% sheep blood agar, eosin methylene blue, and chocolate agar after being centrifuged at 1500-2000 rpm for 15 minutes and were then incubated at 37°C and 5% CO2 for 48 hours. Positive results were identified using Biomerieux Vitek2 (USA). Sediment was also subjected to gram staining. Ziel-Nelson and Indian ink staining were performed for detecting *Mycobacterium* and *Cryptococcus* species, respectively, when they were suspected.

**Data Analysis**

Patients were divided into two groups based on the microbiological results after the neurosurgery: with meningitis (positive CSF culture or smear) and without meningitis. All clinical and laboratory features, including cellular and biochemical indicators of CSF, were compared between the two groups and analyzed statistically using SPSS Version 22 (SPSS Inc., Chicago, IL., USA). The chi-square (𝜒 2) test, independent t-test, and Mann-Whitney U test were used to analyze qualitative and quantitative variables.

## Results

Of 683 patients with neurosurgical procedures, a total of 115 patients were included in this study, of whom 23 patients had positive culture (Table 1) and were categorized as the meningitis group, and 92 patients were in the non-meningitis group. There was no significant difference between the two groups in terms of demographics and underlying diseases. Also, no significant difference was found in the type of surgery and external catheter between the two groups (Table 2).

**Table 1:** Causative pathogens isolated from CSF in the bacterial meningitis group

Isolated pathogens	Frequency	%
*Klebsiella pneumonia*	7	30.4
*Acinetobacter baumannii*	5	21.7
*Escherichia coli*	4	17.4
*Pseudomonas * *aeruginosa*	3	13.0
*Enterobacter aerogenes*	2	8.7
*Enterococcus faecalis*	1	4.3
*Enterococcus faecium*	1	4.3
Total	23	100

**Table 2:** Characteristics of the patients with a history of neurosurgical procedure

	Non-meningitis group (92)	Meningitis group (23)	Total (115)	P-value
Age (mean ± SD)	45.8±17.9	39.9±14.4	41.8±16.7	0.143
Gender N (%)				
Male	36 (39.1)	8 (34.1)	44 (38.2)	0.124
Female	56 (60.9)	15 (65.9)	71 (61.8)	
Underlying diseases n (%)				
Hypertension	4 (4.3)	0 (0)	4 (3.5)	0.341
Ischemic heart diseases	5 (5.4)	0 (0)	5 (4.3)	0.581
Diabetes mellitus	6 (6.5)	1 (4.3)	7 (6.1)	0.999
Hypothyroidism	15 (16.3)	4 (17.4)	19 (16.5)	0.900
Type of neurosurgery				
Transcranial	66 (71.7)	15 (69.6)	81 (70.4)	0.741
Endoscopic transnasal	26 (28.3)	7 (30.4)	33 (28.7)	
External ventricular drain				
Yes	19 (20.7)	6 (27.3)	25 (21.7)	0.500
No	73 (79.3)	17 (32.7)	90 (78.3)	

Table 3 shows the comparison of CSF analyses between the two groups. There was no significant difference in the mean CSF LDH level, CSF/serum LDH ratio, mean CSF protein and mean red blood cells (RBC) between the two groups. However, the mean CSF glucose (*P*=0.029) and CSF/serum glucose ratio (*P*=0.008) were significantly lower in the meningitis group (59.5±33.9 vs. 76.8±37.5 mg/dL and 43.7% vs. 56.3%, respectively). The optimal cut-off point for CSF glucose was 63.5 mg/dL with 62% and 70% sensitivity and specificity, respectively. In comparison, the cut-off point value for the CSF/serum glucose ratio was 35.95% with 85.9% sensitivity and 40% specificity.

The mean CSF white blood cells (WBC) and neutrophil count were significantly elevated in the bacterial meningitis group compared with the other group (4219.1 vs. 1317.3 cells/mL and 68.6% vs. 34.4%, respectively). 

**Table 3:** CSF biological findings in post-neurosurgical patients with and without meningitis

Parameter	Non-meningitis group (92)	Meningitis group (23)	*P*
CSF glucose mg/dL (mean ± SD)	76.8±37.5	59.5±33.9	0.029*
CSF/blood glucose ratio (%)	56.3	43.7	0.008*
CSF Lactate mmol/L (mean ± SD)	2.8±2.3	4.8±2.2	0.381
CSF/blood lactate ratio (%)	30.6	48.5	0.363
Protein mg/dL (mean ± SD)	100.1±85.5	72.2±67.5	0.163
White blood cell count/mm^3^ (mean ± SD)	1317±839	4219±4672	0.001*
Neutrophil dominancy (%)	34.4	68.6	< 0.001*
Red blood cell count/mm^3^	4743±3817	6760±3215	0.161

## Discussion

Our single-center study, which compared the CSF parameters between 23 patients with PNM and 92 post-neurosurgical patients without meningitis, to our knowledge is the first study reporting the value of these indices comparatively. Various studies have not been able to provide specific CSF findings with high positive and negative predictive values for the PNM diagnosis, possibly owing to some conditions, such as hemorrhage and inflammation, occurring in the CSF following the neurosurgical procedures (15-17). Therefore, the number of RBCs in the CSF may not indicate infection (6, 18). In our study, the median number of RBC in the CSF was not significantly different between the two groups although it had no diagnostic value for PNM.

The CSF LDH is elevated following the spread from ischemic tissues or anaerobic metabolism of bacterial infections (19, 20). Study findings support the use of high CSF LDH levels as a laboratory marker for the diagnosis of acute bacterial meningitis (15, 19, 21). However, the results of our investigation did not support the use of CSF LDH level for diagnosis of PNM.

Proteins derived from brain tissues are known to have a higher concentration in the CSF than in the serum (22, 23). Although a significant increase in CSF protein levels has been reported in bacterial meningitis in some studies (24, 25), there was no significant difference in the mean CSF protein between the two groups in our study.

Although a normal range for the CSF glucose has not been defined, studies suggest that the CSF glucose is approximately two-thirds of the serum glucose (26-28). The CSF glucose level decreases in bacterial meningitis. However, the normal level of CSF glucose does not rule out CNS bacterial infections (29, 30). Other conditions, such as chemical meningitis, inflammatory processes, and intracranial hemorrhage can also cause hypoglycorrhachia (26, 31). The findings of a study on patients with PNM revealed that the CSF glucose values were not sufficiently sensitive or specific to diagnose PNM (32). In another study on adults with CSF shunt–associated infections, only slightly more than 50% of patients had a CSF/serum glucose ratio of < 0.5 (15). Our results showed that the CSF glucose level and CSF/serum glucose ratio were significantly lower in the PNM group. Although the normal CSF/serum glucose ratio has not been rigorously investigated, it is cited as 0.6 by the standard reference texts (33). In the current study, the CSF/serum glucose ratio in patients without meningitis was near 0.6 but was significantly lower in the PNM group.

So far, no CSF or blood parameters alone have been introduced for the diagnosis of bacterial meningitis. However, an increased leukocyte count and a relative predominance in CSF neutrophils (>50%) may be diagnostic for bacterial meningitis (17, 34). In a comparative report on the CSF analysis between patients with bacterial meningitis and those without meningitis, the median WBC count (98 vs. 494 cell/mm^3^) and neutrophil count (20 vs. 428 cell/mm^3^) were significantly higher in the group with bacterial meningitis (34). On the contrary in another study, WBC count and neutrophil dominance were among the weakest parameters to differentiate between bacterial and viral meningitis (17). Our results revealed that the mean CSF WBC count and neutrophil count were significantly elevated in the PNM group compared with the patients without meningitis.

The diagnostic value of some inflammatory markers, such as C-reactive protein, tumor necrosis factor-α, procalcitonin, and interleukin-6, in the diagnosis of PNM, has been suggested but not approved yet (6). The main limitation of our study is the lack of examination of these inflammatory factors in the CSF.

## Conclusion

The diagnosis of PNM is challenging in clinical and laboratory settings. Based on our findings, the CSF glucose level of less than 60 mg/dL, CSF/serum glucose ratio of less than 0.44, CSF WBC count of more than 4200 cell/mm^3^, and CSF neutrophil dominancy of more than 70% can be valuable in PNM diagnosis. Other CSF biochemical markers had no contribution to the PNM diagnosis in our study. Future research is needed to examine the value of CSF inflammatory markers in the PNM diagnosis. 
